# Automated Ensemble Modeling with *modelMaGe*: Analyzing Feedback Mechanisms in the Sho1 Branch of the HOG Pathway

**DOI:** 10.1371/journal.pone.0014791

**Published:** 2011-03-30

**Authors:** Jörg Schaber, Max Flöttmann, Jian Li, Carl-Fredrik Tiger, Stefan Hohmann, Edda Klipp

**Affiliations:** 1 Institute for Experimental Internal Medicine, Medical Faculty, Otto von Guericke University, Magdeburg, Germany; 2 Theoretical Biophysics, Department of Biology, Humboldt University, Berlin, Germany; 3 Department of Cell and Molecular Biology, University of Gothenburg, Göteborg, Sweden; Science Commons, United States of America

## Abstract

In systems biology uncertainty about biological processes translates into alternative mathematical model candidates. Here, the goal is to generate, fit and discriminate several candidate models that represent different hypotheses for feedback mechanisms responsible for downregulating the response of the Sho1 branch of the yeast high osmolarity glycerol (HOG) signaling pathway after initial stimulation. Implementing and testing these candidate models by hand is a tedious and error-prone task. Therefore, we automatically generated a set of candidate models of the Sho1 branch with the tool *modelMaGe*. These candidate models are automatically documented, can readily be simulated and fitted automatically to data. A ranking of the models with respect to parsimonious data representation is provided, enabling discrimination between candidate models and the biological hypotheses underlying them. We conclude that a previously published model fitted spurious effects in the data. Moreover, the discrimination analysis suggests that the reported data does not support the conclusion that a desensitization mechanism leads to the rapid attenuation of Hog1 signaling in the Sho1 branch of the HOG pathway. The data rather supports a model where an integrator feedback shuts down the pathway. This conclusion is also supported by dedicated experiments that can exclusively be predicted by those models including an integrator feedback.

*modelMaGe* is an open source project and is distributed under the Gnu General Public License (GPL) and is available from http://modelmage.org.

## Introduction

Dynamic models of complex biochemical networks have become an indispensable tool in biochemical and genetic research [1], [2], [3]. Despite enormous efforts in experimental research in cellular and molecular biology, there is still a substantial uncertainty in both qualitative and quantitative aspects of biochemical networks. These uncertainties need to be resolved by confronting alternative mathematical models with experimental data and by a combination of model selection and parameter fitting [4], [5].

Possible combinations of uncertain structures and kinetics directly translate into alternative mathematical models. Generating and managing such candidate models poses a considerable challenge to the modeler. This is mainly because of the combinatorial complexity of model alternatives that often renders it a tedious and error-prone task to implement and handle each model individually. Currently, there is no tool that automatically generates, implements, manages and discriminates a specific user-defined set of candidate models that differ in both structure and kinetics.

Another debated issue is model documentation [6], [7]. It is not only the successful models that are of interest to the research community, but also those that failed. Usually, in the course of a modeling project many unsuccessful model versions are tested but only the successful one is finally published. The unsuccessful versions, even though of interest, are never documented, because such documentation is a laborious task and unrewarding task not rewarded.

In order to handle uncertainty in kinetics and model structure, we developed the tool *modelMaGe* that automatically generates candidate models based on a single master model and specified modifications [8]. The generated models are automatically documented such that it is always apparent how they were derived from the master model, thereby keeping track of model alternatives. Finally, all generated models are automatically simulated, fitted to data (if available), and compared. At the end the user is provided with a ranking of the model fits and statistical measures that enable him to discriminate between model alternatives.

The aim of this study was to elucidate which mechanism(s) could be responsible for shutting down the response of the Sho1 branch of the high osmolarity glycerol (HOG) signaling pathway in yeast, a question that was also addressed in a recent paper [9]. In this paper, the authors compared five different models, each employing a different negative feedback mechanism. In all models the activated Hog1 kinase exerts a negative feedback onto its own activation by deactivating upstream components. The model that fitted the data best included a Hog1-mediated desensitization of Sho1, an upstream membrane protein that interacts with the putative receptors of the pathway [10]. Subsequently, it was shown by experiments that Hog1 phosphorylates Sho1, suggesting that the phosphorylated form of Sho1 displays diminished signaling capacity. This would result in the negative feedback loop suggested by the model and rapid attenuation of Hog1 signaling.

There are, however, experimental observations and theoretical considerations that argue against such a scenario. It is well known that the HOG pathway is a perfect adaptor: following adaptation to high osmolarity the signaling pathway is shut off [2], [11], [12], [13] and phosphorylated Hog1 levels return to the pre-stress situation. From theory it follows that perfect adaptation is impossible in a signaling pathway with a constant signal and a negative feedback of a downstream component to an upstream component. The result will always be either a non-zero steady state or oscillations, either damped or sustained [14], [15], [16]. In a recent study on simplified signaling networks it was shown that there are in principle two mechanisms that can bring about perfect adaptation [17], a negative integrator feedback [11], [13], [18] or an incoherent feed-forward loop [19]. In the HOG pathway adaptation is supposedly due to an integrator feedback control, consisting of the accumulation of intracellular glycerol, which balances the osmotic pressure gradient imposed by an osmotic shock [2], [11], [20]. However, most studies studying the adaptation mechanisms in baker's yeast concentrated on the wild-type yeast [11], [13] or on the Sln1-1 branch [2].

The aim of this study is to systematically explore several hypotheses for the feedback mechanisms in the Sho1 branch of the HOG pathway and test which of those are best supported by the data published by Hao et al. (2007) with by a model discrimination analysis. This endeavor is largely facilitated by the use of the tool *modelMaGe*. Our analysis suggests that the reported data does not support the conclusion that a negative feedback of activated Hog1 on the upstream Sho1 leads to rapid attenuation of Hog1 signaling. The data rather supports a model where an integrator feedback shuts down the pathway. This conclusion is also supported by dedicated triple osmo-shock experiments that can exclusively be predicted by those models including an integrator feedback.

## Results

### The candidate models

**Figure 1:**
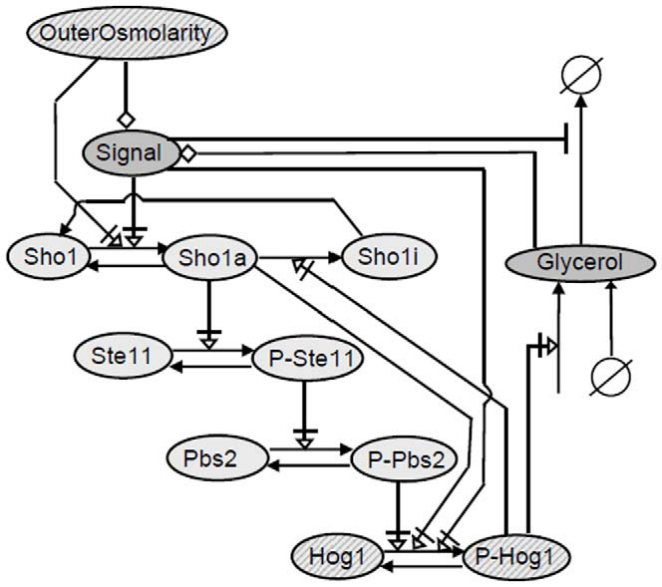
The wiring scheme of the master model, including all components and reactions of the potential candidate models in SBGN. Light gray indicate components of the original model Ca10 by Hao et al. (2007) (Table 1). Dark gray components indicate components of the C5 model (Table 1). Hatched components are part of both models.

We developed a master model that includes the best fitting model of Hao et al. (2007) as well as a set of other candidate models. In line with the purpose of this study, we keep the models as simple as possible and abstract from concepts like volume change, turgor, transcription, etc. that are known to be involved in HOG signaling and osmo-adaptation [2]. The wiring diagram of the master model is depicted in Figure 1 in Systems Biology Graphical Notation (SBGN) [21].

In short, osmo-adaptation in yeast by the Sho1 branch of the HOG pathway functions through activation of several membrane proteins involving Sho1 [10] that trigger a mitogen activated protein (MAP) kinase cascade, including Ste11, Pbs2 and Hog1. Activated Hog1 either directly by increasing metabolic fluxes or indirectly via a transcriptional response stimulates glycerol production to balance the water potential differences between inside and outside of the cell thereby recovering the pre-shock volume [12].

As indicated in the introduction, the main new feature we wanted to test in order to explain the data is a negative feedback that involved an integral response instead of a transient response (P-Hog1-mediated conversion of active Sho1 (*Sho1a*) to desensitized Sho1 (*Shoi*) (Figure 1, reaction *v3* in Figure S1 in Supporting Information S1)). We achieved this by assuming that phosphorylated, i.e. activated, Hog1 (*P-Hog1*) stimulates the production of intracellular glycerol (Figure 1, reaction *v11* in Figure S1 in Supporting Information S1). The newly introduced component *Signal* mimics the notion that it is the imbalance of internal and external water potential (for simplicity represented by *Glycerol* and *Outerosmolarity*, respectively), that activates the signaling pathway, rather than just the external osmolarity. Therefore, *Signal* is defined as the difference between *OuterOsmolarity* and *Glycerol*. Accumulation of *Glycerol* can also be achieved by constitutive production of glycerol and impaired outflow through closure of the glycerol channel Fps1, which is also subject to regulation (here by *Signal*) (Figure 1, reactions *v12* and *v13* in Figure S1 in Supporting Information S1) [12], [22].

**Figure 2:**
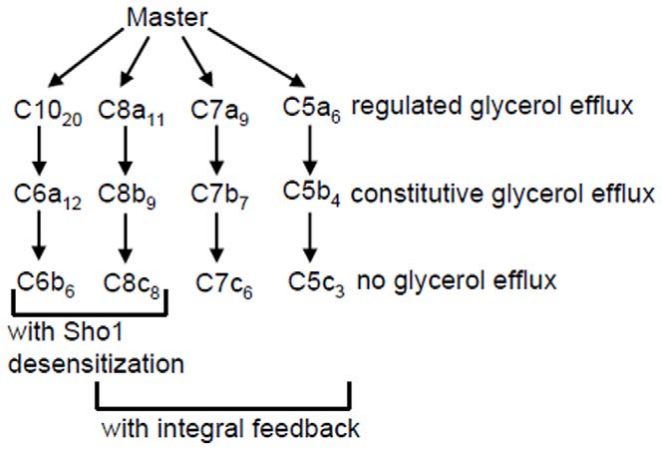
Model tree. Schematic representation of the generated candidate models and their features. Models are named according to their number of species. The numbers in the subscript indicate the number of fitted parameters.

We systematically tested various combinations of these different feedback mechanisms, which are depicted in a model tree in Figure 2. For simplicity, we name the generated models according to their number of species.

The candidate models in the leftmost branch are the original model published by Hao et al. (2007) (*C10*) and simplifications thereof. Simplifications are achieved by leaving out components and/or using simpler reaction kinetics. The two leftmost branches include the feedback where *P-Hog1* mediates conversion of active Sho1 (*Sho1a*) into inactive Sho1 (*Sho1i*) (Sho1 desensitization, Figure 1). The three rightmost branches include the integral feedback, where pathway activation is regulated by *Signal* as described above. The three rightmost branches vary in their number of intermediate signaling components with the simplest model *C5* only having five components (Figure 1). The respective simplifications of the models in the three rightmost branches concern assumption about the glycerol accumulation. They either have a regulated glycerol efflux, including *P-Hog1* activated and constitutive glycerol production, a constitutive, i.e. non-regulated, glycerol efflux, including only *P-Hog1* activated glycerol production or no glycerol efflux, also including only *P-Hog1* activated glycerol production. The latter corresponds to the hypothesis that the glycerol channel quickly closes and does not open again in the simulated time frame. Detailed wiring schemes of the master model and all candidate models are shown Figures S2-S13 in Supporting Information S1.

### Candidate Model Generation and Discrimination

The candidates were automatically generated by *modelMaGe*, to which we only provided the master model (Figure 1), and the directives specifying which components should be removed for each candidate model and which kinetics should be used. The master model is formulated in *Copasi*-format, because the parameter estimation task also has to be specified, when the candidate models are supposed to be fitted to data (see Methods section). Model generation, fitting and ranking is then automatically performed by *modelMaGe* using *Copasi* as the simulation engine by a single command (see Supporting Information S1). The master model, the directives for *modelMaGe*, the data and other details are supplied in Supporting Information S1. The ranking of the candidate models according Akaike Information Criterion corrected for small sample sizes (AICc) is displayed in Table 1.

**Table 1:** Model ranking.

Rank	Model	k	SSR	AICc	feedback	Hog1-PSS
1.	C5c	3	0.251	-38.045	I	<0.05
2.	C5b	4	0.251	-34.104	I	<0.05
3.	C7c	5	0.259	-31.316	I	<0.05
4.	C6a	12	0.061	-29.246	D	>0.05
5.	C7b	6	0.258	-27.373	I	<0.05
6.	C7a	9	0.153	-26.465	I	<0.05
7.	C5a	7	0.241	-25.091	I	<0.05
8.	C8c	7	0.259	-23.335	D+I	<0.05
9.	C8b	8	0.258	-19.393	D+I	<0.05
10.	C8a	11	0.153	-14.537	D+I	<0.05
11.	C6b	6	0.740	-1.069	D	>0.05
12.	C10	20	0.049	164.842	D	>0.05
	Hao	20	0.181	205.92	D	>0.05

*k*: number of parameters. *SSR*: sum of squared residuals as calculated by Copasi, *AICc*: Akaike Information Criterion corrected for small sample size. *n* is 25 for all models. In the last line the SSR and the corresponding AICc of the original model (C10) with the parameter set from Hao et al. (2007) is displayed. *feedback*: the type of feedback employed by the model (D: Sho1 desensitization, I: integrator feedback).

**Figure 3:**
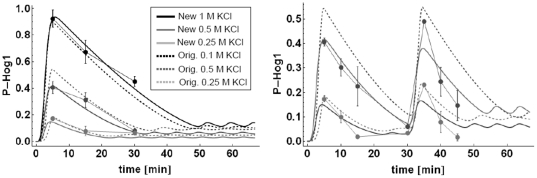
Time course simulations of P-Hog1 for single (t = 0) and double (t = 0,t = 30 min) osmotic shocks of different concentrations for the *C10* model, both with original parameters from *Hao* et al. (2008) (dashed lines, Orig.) and re-fitted parameters (lines, New).

In terms of accuracy of the fit (*SSR*) the model by Hao et al. (2007) both in its original form as well as in the simplified version with Michaelis-Menten kinetics (*C10* and *C6a*) performed best. In fact, the fits are even better than with the parameter set from the original publication (last line in Table 1). However, *C10* is ranked lowest according to the AICc, because of its high number of parameters. Thus, in terms of parsimonious representation of the data it performed worst. Time course simulations of the original model *C10* with the original parameter set from Hao et al. (2007) showed damped oscillations in the *P-Hog1* concentrations (Figure 3). The *C10* model with the new parameter sets converges to sustained oscillations around a steady state, which both with the original parameter as well as with the newly fitted parameters increased with increasing osmotic shock (Figure 3), as expected from theory.

**Figure 4:**
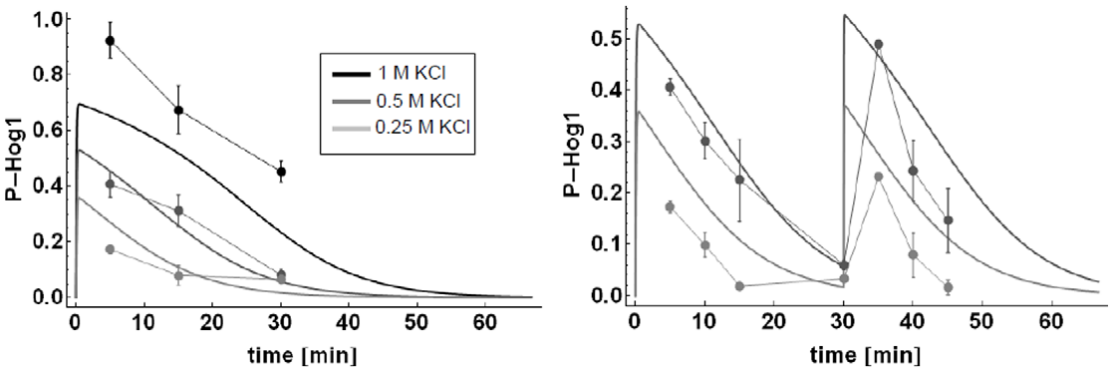
Time course simulations of P-Hog1 for single (t = 0) and double (t = 0,t = 30 min) osmotic shocks of different concentrations for the *C5c* model.

Recent publications on the Hog1 dynamics upon osmotic shock in yeast with a much higher time resolution [11], [13], [23] imply that oscillations as well as increasing steady state concentrations are spurious effects and features that are not present in the data. Fitting spurious effects in the data is an indication of an over-fitted model. The most prominent dynamic feature of the *P-Hog1* time series, i.e. a rapid increase and slower decline to the initial state, can faithfully be captured by the most simple three-parameter model *C5c* (Figure 4). In terms of parsimonious representation of the data (*AICc*) this model is ranked highest.

To challenge a critical qualitative property, we tested which of the model candidates did or did not show perfect adaptation behavior by comparing initial and steady-state simulated Hog1 activation after adaptation. Models were considered not to show perfect adaptation when their simulated steady-state value of *P-Hog1* one hour after stimulation was above 5% the total protein concentration. We employed the 5% threshold, because we consider this value close to the measurement error, i.e. a measured value of 5% of the maximum is practically zero. Therefore, we treated simulated values below 5% of the possible maximum as zero and therefore perfectly adapted. Strikingly, only those models that did not include an integrator feedback (*C10*, *C6a*, *C6b*) were not able to show perfect adaptation according to this criterion.

### Model Predictions for Triple Shock

**Figure 5:**
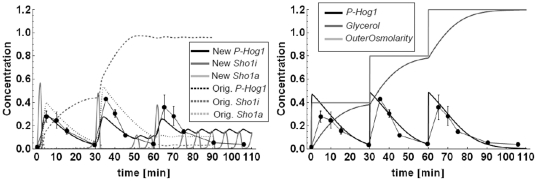
Triple shock (t = 0, t = 30 min, t = 60 min) predictions and data for the *C10* model (left panel, *New*: with newly estimated parameters, *Orig*.: with the original parameters from [**9**] **) and the** ***C5c*** **model (right panel).** The maximum of the 0.4 M KCl triple shock time series is scaled to the maximum of the 1 M KCl single shock time series. The error bars represent the standard deviation of three independent measurements. For pictures of the original Western Blots please refer to Figure S14 in Supporting Information S1.

Over-fitted models, even though they tend to identify spurious effects are often better in predictions than under-fitted models [24]. We tested whether the simple *C5c* model was under-fitted by predicting and comparing simulations to additionally measured data of *P-Hog*1 time courses after repeated osmotic shock with 0.4 M KCl for both, *C5c* and the *C10* model (Figure 5). The amount of KCl was added to the culture three times with 30 minutes intervals.

Upon triple shock, the *C5c* model replicated the single shock *P-Hog1* profile a third time, as it is also seen in the data. The *C10* model with the original parameter set showed no Hog1 activation upon a third consecutive shock. This can be explained by the fact that all activated receptor protein Sho1 (*Sho1a*) was already desensitized after the second shock (*Sho1i,* light gray dashed line in Figure 5) and not yet recycled again, in order to be able to react to a third shock (dark gray dashed line in Figure 5). The *C10* model with the new parameter set was able to show a third P-Hog1 response. However, the third response was weakened and again resulted in sustained oscillations around an even higher steady state concentration. Interestingly, with the new parameter set the model *C10* was only able to react to a third shock at the expense of the desensitization mechanism of activated Sho1 (*Sho1a*), i.e. *Sho1i* showed no response at all (light gray curve in Figure 5). In fact, the velocity of the reaction that facilitated the conversion of *Sho1a* to *Sho1i* was at the lower boundary allowed in the parameter estimation (10^−6^, Supporting Information S1) and therefore negligible. The time courses of *Sho1a* showed an oscillatory behavior (dark gray curve in Figure 5) corresponding to the oscillations in *P-Hog1*.

## Discussion

The aim of this study was to analyze feedback mechanisms in the Sho1 branch of the HOG pathway that are best supported by a data set of the dynamics of *P-Hog1* upon single and double shock. The use of *modelMaGe* allowed us to systematically explore an ensemble of model candidates, also documenting unsuccessful candidates. The results are completely transparent, comprehensible and easily communicated to the community, as the master model, the data, as well as the directives how to generate candidate models are described in a compact and comprehensible manner. Moreover, the fitting and ranking procedure can be reproduced online at http://modelmage.org using the master model and the reduction directives provided in Supporting Information S1.

The generated models comprised the best model of Hao et al. (2007) as well as other alternatives including several types of transient and/or integrator feedbacks. The set of candidate models was automatically generated and fitted to data given in Hao et al. (2007). In addition, *modelMaGe* automatically generated a ranking of the fitted models according to the Akaike Information Criterion corrected for small sample size (*AICc*).

We show that according to the *AICc* the three-parameter *C5* model approximates the data better in terms of parsimony than the 20-parameter *C10* model. The original model seems to fit spurious effects in the data, indicating that it was over-fitted. Instead, our parsimonious three-parameter model could predict the triple shock Hog1 activation profile better than the *C10* model with the original parameter set. We found also a new parameter set for the original *C10* model that fitted the data best, but was ranked worst according to the *AICc*, because of its high number of parameters. The *C10* model with the new parameter set was able to predict the triple shock Hog1 activation profile, but only at the expense of the feedback mechanism that was actually proposed. Therefore, we conclude that even though Hao et al. (2007) show that Hog1 phosphorylates Sho1 and thereby dampens its own response, the single- and double-shock data they provide do not support the hypothesis that it is this desensitisation mechanism which leads to rapid attenuation of Hog1 signaling in the Sho1 branch. Our model discrimination analysis rather supports the hypothesis that there is a negative integrator feedback acting through glycerol accumulation. This could be tested by measuring internal glycerol concentration for the Ssk2/22 mutant as it has been done for the wild type [2], however, this is out of the scope of our study. Glycerol accumulation mediating adaptation and Hog1 de-activation probably acts via removal of the stimulus, which in turn might be volume or membrane related, e.g. turgor pressure [25]. It has been shown that for the wild type and the Sln1 branch of the HOG pathway that such an integrator feedback are probably responsible for the adaptation response. Here, we provide computational as well as experimental evidence that this is also the case for the Sho1 branch [2], [11], [12], [13]. The rapid attenuation of the signal indicates that there is not necessarily a transcriptional-translational response involved. It has been suggested that this fast integrator feedback by fast accumulation of glycerol can be achieved by a fast activation of glycerol production that does not involve a transcriptional-translational response and/or by rapid closure of the glycerol channel Fps1 [2], [11]. Indeed, the simple *C5c* model that does not include glycerol efflux can be interpreted with both an activation of glycerol production as well as fast closure of the glycerol channel. However, we do not refute that the proposed negative feedback of Hog1 onto Sho1 modulates the Hog1 response and may serve other functions than Hog1 deactivation, e.g. stability of the response, noise filtering, inhibiting crosstalk to other pathways or dose-response alignment, as suggested for the pheromone pathway [26].

We also conclude that *modelMaGe* is a useful tool that facilitates systematic testing a set of candidate models, making the modeling process and its results transparent to the community in an easy and comprehensible manner.

## Methods

### Model generation and discrimination

**Figure 6:**
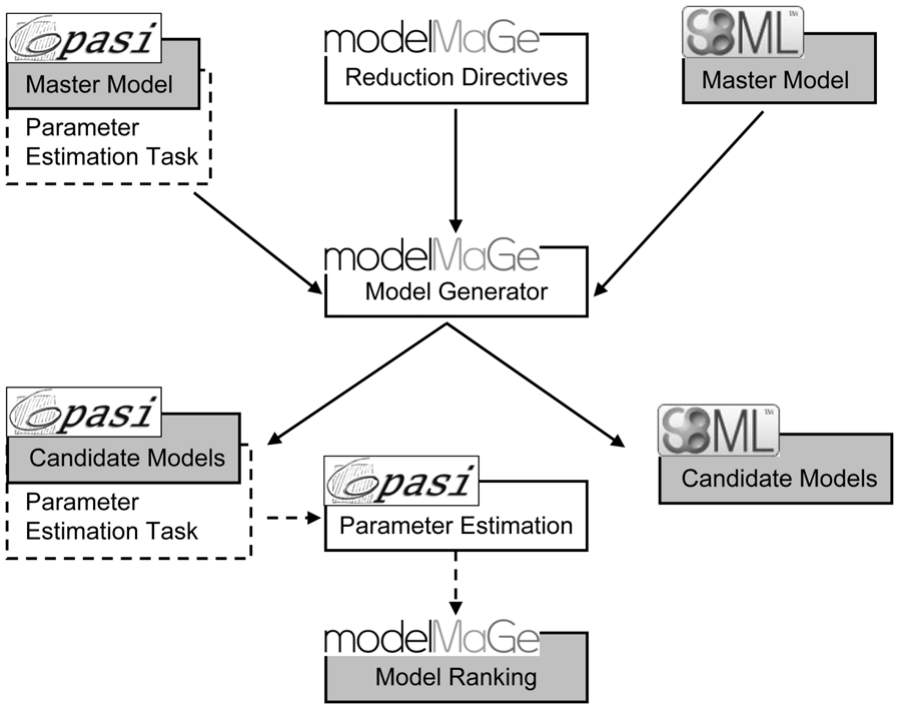
Workflow of *modelMaGe*. Light boxes indicated program tasks that are executed by the programs indicated in the attached boxes. Grey boxes indicate results files, with the respective formats indicated in the attached boxes. The user provides an SBML or *Copasi* file and directives for model generation. *modelMaGe* creates a set of models both on Copasi and SBML format. In case a parameter estimation task was specified in the *Copasi* master model (dashed box) and data is available the generated *Copasi* model are fitted to the data, after which the results are returned to *modelMaGe* that generates a ranking of the candidate models.

The main idea of *modelMaGe* is simple: model alternatives are generated from a master model that includes all alternatives of interest. The master model is the only place that is meant to be manipulated by the modeler, which avoids errors that are introduced by handling several models at the same time. The general workflow is depicted in Figure 6.

Generation of candidate models in *modelMaGe* is a two step process. The first step is to create a master model in *Copasi* [27] or in any other SBML [28], [29] compliant editor like *CellDesigner* [30] or *SemanticSBML* [31]. The master model is a combination of all candidate models that are to be generated and simulated. Thus, the master model must include all possible species and reactions that shall be included in any of the candidate models. In the second step, the set of candidate models is generated by removing reactions, species or modifiers and combinations thereof from the master model and/or by assigning alternative kinetics to certain reactions. The removal of components and exchange of kinetics is done by giving simple logical directives to the program. Details of the usage, technology and algorithms are described in Flöttmann et al. (2008) and at www.modelMaGe.org.

The generated models come as a set of both SBML and *Copasi* files that can readily be simulated by appropriate tools, e.g. *Copasi* or *CellDesigner* (Figure 6). When data for certain components is available, *modelMaGe* can automatically fit the models to the data by estimating parameters. For simulation and parameter estimation *ModelMaGe* utilizes the COPASI simulation engine *CopasiSE*. The parameter estimation task is most conveniently defined in *Copasi*'s graphical user interface. The user has to set up the parameter estimation task only once for the master model. *modelMaGe* automatically defines the parameter estimation task for all generated candidate models. Using the results of the parameter estimation, *modelMaGe* computes the Akaike Information Criterion corrected for small sample sizes (AICc) [24] for each candidate model:
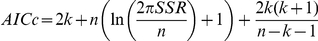
where *SSR* is sum of squared residuals, *k* the number of parameters and *n* the number of data points. The AICc is an information-theory based measure of parsimonious data representation that incorporates the goodness of the fit (*SSR*) as well as the complexity of the model (*k*) and is used to rank the candidate models, thereby giving an objective measure for model selection and discrimination. There also exists a web-based version of *modelMaGe* (http://modelmage.org). For a detailed discussion on the AIC and its usage in model discrimination please refer to [24].

### Comparison between model simulation and data

The measured data is scaled relative to maximal measured value + standard deviation and therefore has arbitrary units. Accordingly, for the simulated values of phosphorylated Hog1 an assumption has to be made what percentage of the total Hog1 is phosphorylated upon maximal phosphorylation. For simplicity, we assumed that maximally 100% of the total Hog1 can be phosphorylated. As can be seen in Figure 3 and 4 this assumption fits nicely to that data. In fact, the measured maximum scaled value of *P-Hog1* was 0.92 (Figure 3 and 4) and it is known that a) only the phosphorylated form enters the nucleus and b) upon strong stimulation almost all Hog1 enters the nucleus [32]. Therefore, it is a reasonable model result that upon stimulation with 1 M KCl around 90% of the total Hog1 becomes phosphorylated.

### Western blotting

*Saccharomyces cerevisiae* cells BY4741 ssk1Δ (BY4741; Mat a; his3Δ1; leu2Δ0; met15Δ0; ura3Δ0; ssk1::kanMX4, from the Saccharomyces Genome Deletion Project) were grown in synthetic complete medium (1x Difco™ YNB base, 1x Formedium™ Complete Supplement Mixture, 0.5% ammonium sulfate, 2% glucose) on a rotary shaker at 225 rpm at 30°C until reaching an optical density of 1.0 measured at 600 nm. Cells were osmotically shocked as noted with KCl from a 4M stock solution. Samples of 1 ml were taken and cells harvested by centrifugation at 14000 rpm for 30 s and the pellet frozen in liquid nitrogen. Times given in the data are the times of freezing. Total protein extracts were made from the frozen cell pellets by boiling for 6 min in 60 µl extraction buffer (Tris-HCl 75 mM pH 6.8, Glycerol 15%, DTT 150 mM, SDS 3%, NaF 8 mM, Na_3_VO_4_ 75 µM, β-mercaptoethanol 0.11%). Protein samples were separated using SDS-PAGE (Tris-Cl) and transferred to nitrocellulose. Phosphorylated and total amounts of Hog1 protein were detected using antibodies #9211(Cell-Signaling Technology) and #yC-20(Santa Cruz Biotechnology) respectively. The membranes were processed for infrared fluorescent detection using secondary antibodies #926-32223(LI-COR biosciences) and #926-32214(LI-COR biosciences) respectively, and scanned for both fluorescent channels using an ODYSSEY IR-scanner(LI-COR biosciences). The signal from phosphorylated Hog1 was divided with the total Hog1 protein signal. The measurements were repeated three times with independent cell cultures (Figure S14 in Supporting Information S1).

## Supporting Information

Supporting Information S1 The supporting information, including supplementary figures.
(0.74 MB DOC)
