# Supplementary material for: Automated Ensemble Modeling with modelMaGe: Analyzing Feedback Mechanisms in the Sho1 Branch of the HOG Pathway
Source: PLoS One. 2011 Mar 30;6(3):e14791. doi: 10.1371/journal.pone.0014791 (PMC3068199; doi:10.1371/journal.pone.0014791)
Supplement: Supporting Information S1 — The supporting information, including supplementary figures. (0.74 MB DOC) [file pone.0014791.s001.doc]

Automated ensemble modeling with *modelMaGe*: analyzing feedback mechanisms in the Sho1 branch of the HOG pathway

# Supporting Information

Jörg Schaber^1,2*^, Max Flöttmann^2^, Jian Li^2^, Carl-Fredrik Tiger^3^, Stefan Hohmann^3^, Edda Klipp^2*^

^1^ Institute for Experimental Internal Medicine, Medical Faculty, Otto von Guericke University, Magdeburg, Germany

^2^ Theoretical Biophysics, Department of Biology, Humboldt University, Berlin, Germany

^3^ Department of Cell and Molecular Biology, University of Gothenburg, Göteborg, Sweden

^*^ To whom correspondence should be addressed:

Jörg Schaber

Institute of Experimental Internal Medicine

Medical Faculty

Otto von Guericke University

Leipziger Str. 44, 39120 Magdeburg, Germany

Phone: +49 391 67 14453

Fax: +49 391 67 13312

Email: [schaber@med.ovgu.de](mailto:schaber@med.ovgu.de)

Model generation

The set of candidate models are automatically generated, fitted and discriminated by *modelMaGe*. Having *modelMaGe* and its prerequisite software installed as described in the documentation at [www.modelmage.org](http://www.modelmage.org/), this achieved by a single simple command:

modelmage.py –p –i Sho1.ini Sho1Master.cps

The –p option indicates that the generated models shall be fitted according to the parameter estimation task that is defined in the master model Sho1Master.cps.

The file Sho1.ini specifies how the candidate models are generated from the master model by leaving out components as species, reactions and modifiers and by inserting alternative kinetics for certain reactions.

Both the master model Sho1Master.cps as well the specifications file Sho1.ini can be downloaded from the journal’s website.

# The models

In the following, we provide diagrams of all generated candidate models. The models are displayed as bipartide graphs. Arrows indicate substrates and products of reactions, end-dotted lines indicate modifiers of reactions. MM-tags and MA-tags at the reactions indicate whether Michaelis-Menten kinetic or mass-action kinetic is used, respectively. Note that in the model diagrams the names of the components are displayed, whereas the generation directives operate on identifiers, which are defined in the corresponding SBML file. A mapping of names and identifiers can be displayed with modelMaGe with the –s option.

**
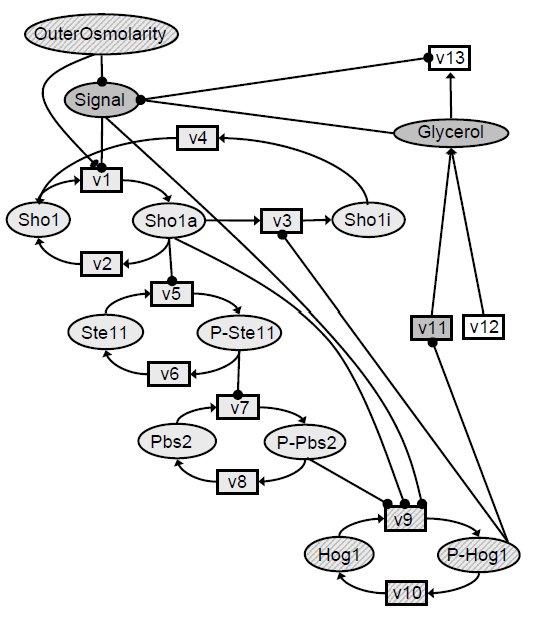
**

**Figure S1**: The master model, including all components (ovals) and reactions (rectangles) of the potential candidate models. The model is depicted as a bipartide graph. Species and reactions are named as in the corresponding Copasi file. This is the data structure *modelMaGe* uses for model generation. Arrows indicate substrates and products of reactions, end-dotted lines indicate modifiers of reaction. Light gray indicate components of the original model *C10* by Hao et al. (2007) (Table 1). Dark gray components indicate components of the *C5c* model (Table 1). Hatched components are part of both models.


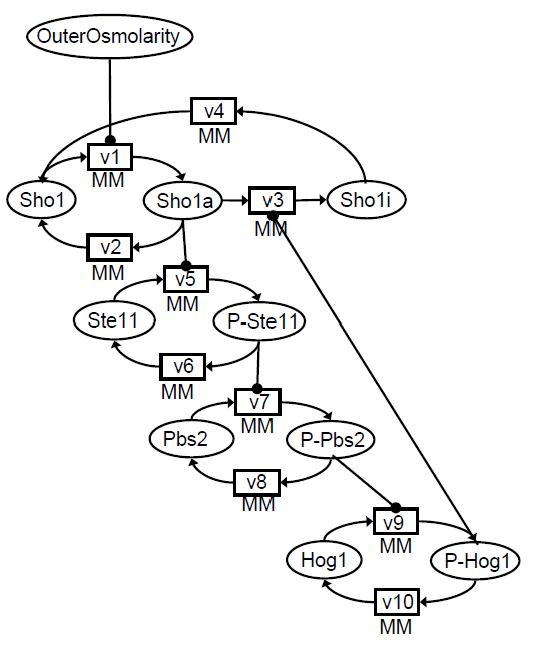


**Figure S2**: The *C10*  model, originally published by Hao et al. (2007) (Model IIIa). The model is generated from the master model by the command: modelmage.py -r ‘species_11 & species_12 & reaction_9:species_3’ –k ‘reaction_1(mMM) reaction_9(mMM)‘ Sho1Master.cps


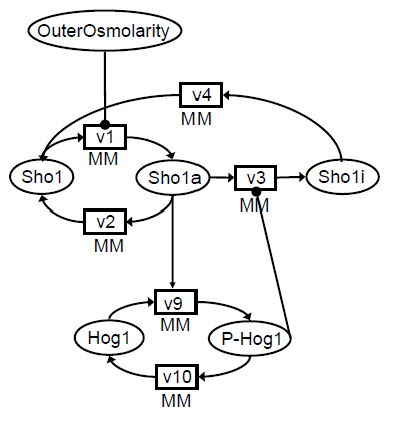


**Figure S3**: The *C6a* model. The model is generated from the master model by the command: modelmage.py -r ‘species_5 & species_6 & species_7 & species_8 & species_11 & species_12’ –k ‘reaction_1(mMM) reaction_9(mMM)’ Sho1Master.cps

**
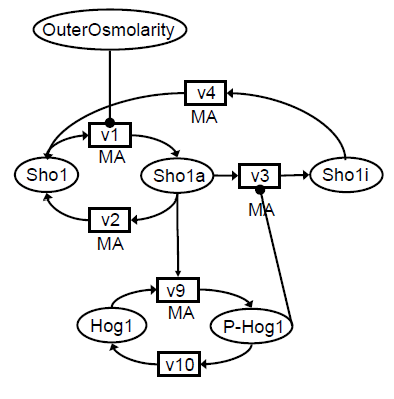
**

**Figure S4**: The *C6b* model. The model is generated from the master model by the command: modelmage.py -r ‘species_5 & species_6 & species_7 & species_8 & species_11 & reaction_11 & reaction_12 & reaction_13’-k ‘reaction_2(MA) reaction_3(mMA) reaction_4(MA) reaction_10(MA)’ Sho1Master.cps


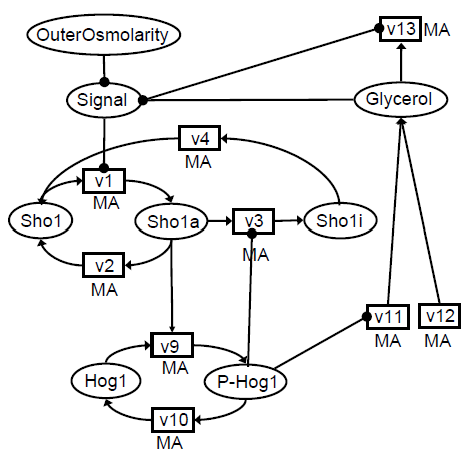


**Figure S5**: The *C8a* model. The model is generated from the master model by the command: modelmage.py -r ‘species_5 & species_6 & species_7 & species_8 & reaction_1:species_1 & reaction_9:species_11’-k ‘reaction_2(MA) reaction_3(mMA) reaction_4(MA) reaction_10(MA)’ Sho1Master.cps


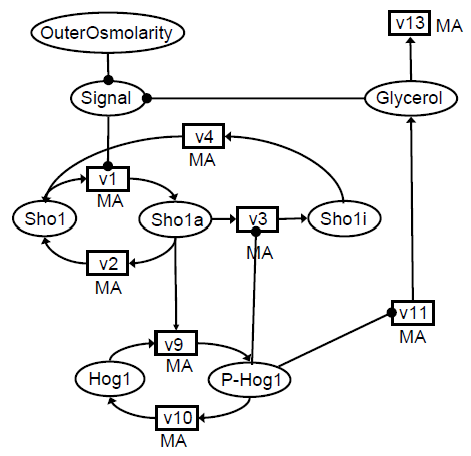


**Figure S6**: The *C8b* model. The model is generated from the master model by the command: modelmage.py -r ‘species_5 & species_6 & species_7 & species_8 & reaction_1:species_1 & reaction_9:species_11 & reaction_13:species_11 & reaction_12’-k ‘reaction_2(MA) reaction_3(mMA) reaction_4(MA) reaction_10(MA)’ Sho1Master.cps


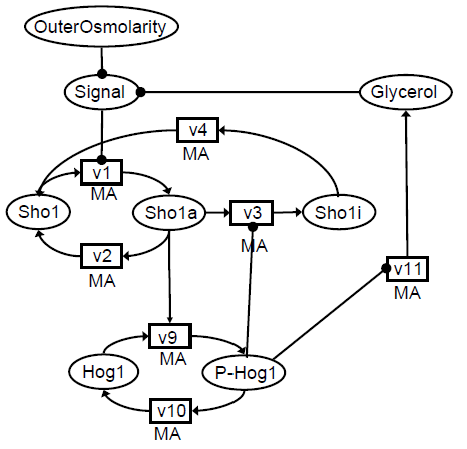


**Figure S7**: The *C8c* model. The model is generated from the master model by the command: modelmage.py -r ‘species_5 & species_6 & species_7 & species_8 & reaction_1:species_1 & reaction_9:species_11 & reaction_12 & reaction_13’-k ‘reaction_2(MA) reaction_3(mMA) reaction_4(MA) reaction_10(MA)’ Sho1Master.cps


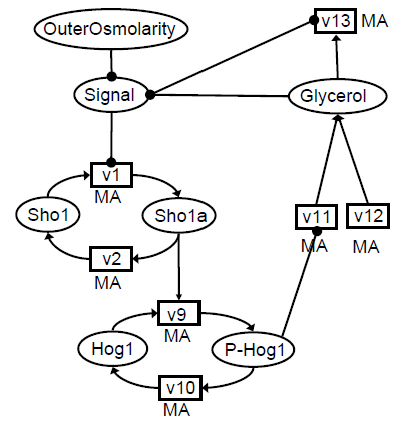


**Figure S8**: The *C7a* model. The model is generated from the master model by the command: modelmage.py -r ‘species_5 & species_6 & species_7 & species_8 & reaction_3 & reaction_4 & reaction_1:species_1 & reaction_9:species_11’ –k ‘reaction_2(MA) reaction_10(MA)’ Sho1Master.cps


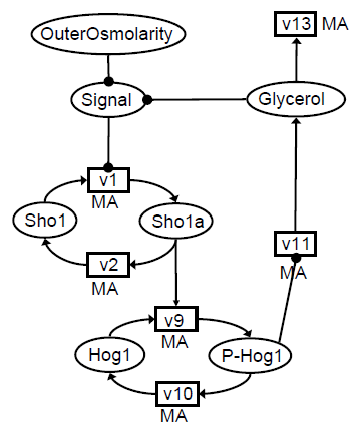


**Figure S9**: The *C7b* model. The model is generated from the master model by the command: modelmage.py -r ‘species_5 & species_6 & species_7 & species_8 & reaction_3 & reaction_4 & reaction_1:species_1 & reaction_9:species_11 & reaction_13:species_11 & reaction_12’ –k ‘reaction_2(MA) reaction_10(MA)’ Sho1Master.cps


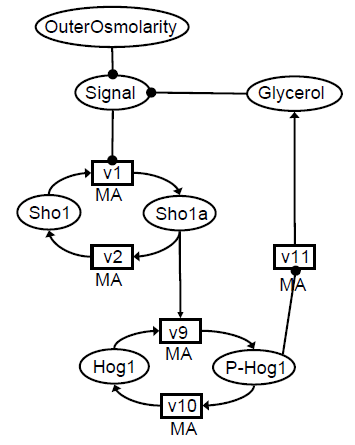


**Figure S10**: The *C7c* model. The model is generated from the master model by the command: modelmage.py -r ‘species_5 & species_6 & species_7 & species_8 & reaction_3 & reaction_4 & reaction_1:species_1 & reaction_9:species_11 & reaction_12 & reaction_13’ –k ‘reaction_2(MA) reaction_10(MA)’ Sho1Master.cps


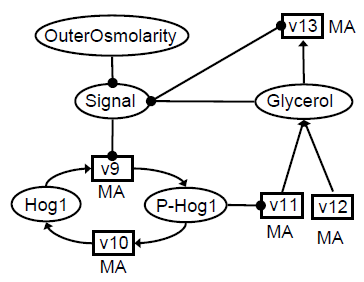


**Figure S11**: The *C5a* model. The model is generated from the master model by the command: modelmage.py -r ‘species_2 & species_3 & species_4 & species_5 & species_6 & species_7 & species_8’ –k ‘reaction_10(MA)’ Sho1Master.cps


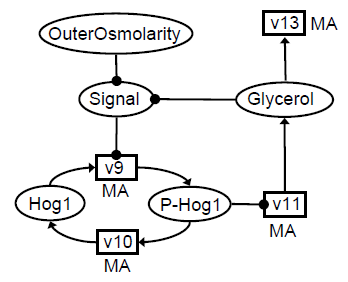


**Figure S12**: The *C5b* model. The model is generated from the master model by the command: modelmage.py -r ‘species_2 & species_3 & species_4 & species_5 & species_6 & species_7 & species_8 & reaction_13:species_11 & reaction_12’ –k ‘reaction_10(MA)’ Sho1Master.cps


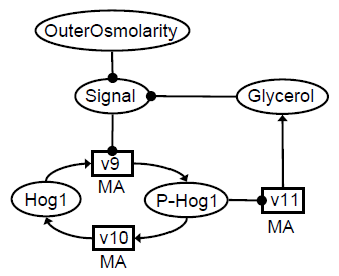


**Figure S13**: The *C5c* model. The model is generated from the master model by the command: modelmage.py -r ‘species_2 & species_3 & species_4 & species_5 & species_6 & species_7 & species_8 & reaction_12 & reaction_13’ –k ‘reaction_10(MA)’ Sho1Master.cps


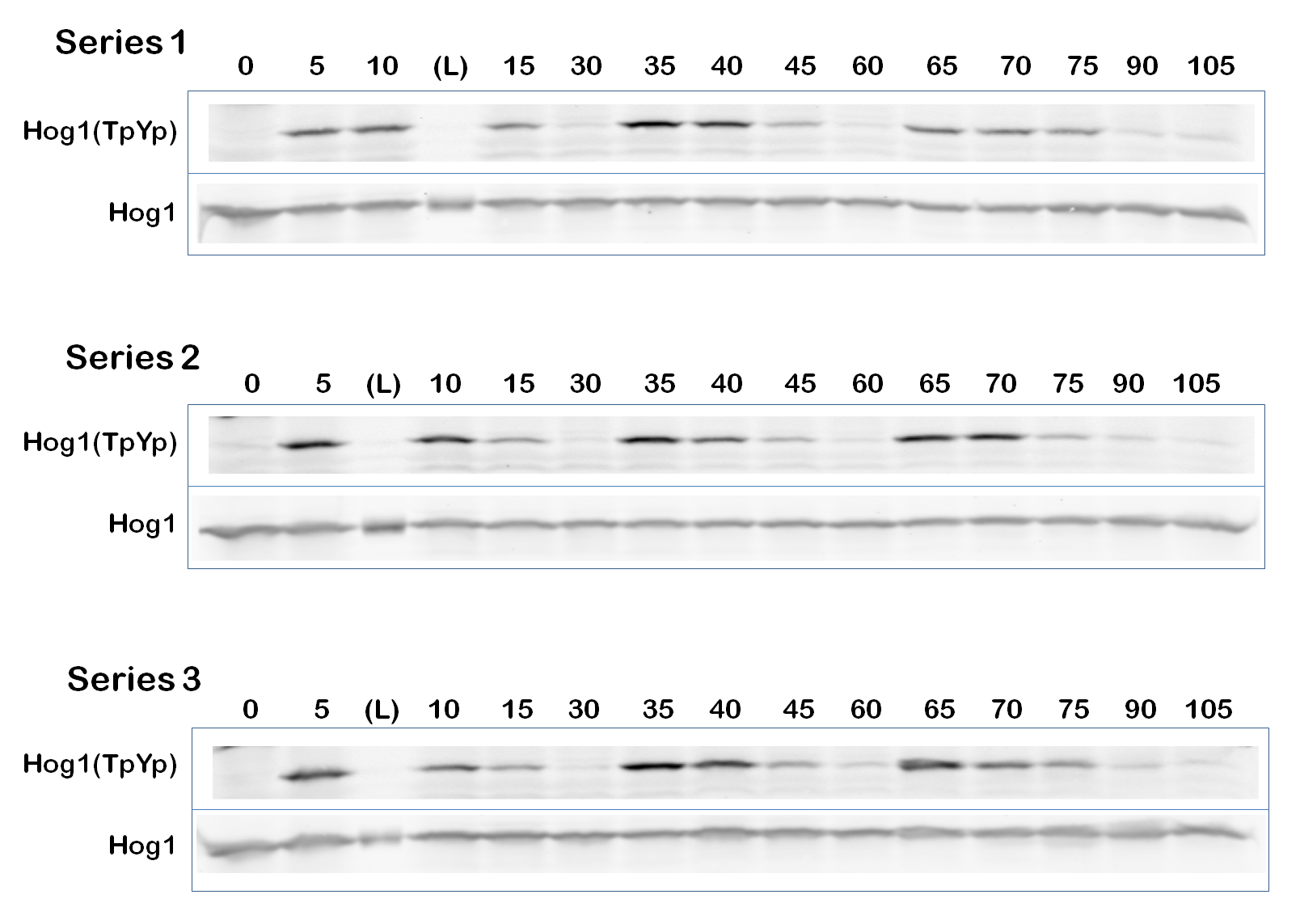


**Figure S14**: Western Blots of the triple shock experiments (t=0, t=30min, t=60min, 0.4 M KCl each). Pictures of the three different experimental data sets (taken from the same membrane). The phospho Thr/Tyr Hog1 signal (Hog1(TpYp) and the total Hog1 protein signal (Hog1) is shown. Lanes are named according to sample times in minutes. (L) are lanes used for protein size ladder (the 50 kD band is visible on the total Hog1 pictures).
